# Psychosocial consequences of the COVID-19 social isolation in the italian general population: Preliminary results

**DOI:** 10.1192/j.eurpsy.2021.837

**Published:** 2021-08-13

**Authors:** A. Ciaramella, M. Rossi, M. Jarego, A. Ferreira-Valente

**Affiliations:** 1 Gift Institute Of Integrative Medicine, Psychosomatic Center, Pisa, Italy; 2 Education Programme Partner With University Of Pisa, Florence, Padua, Miur, Italy, Aplysia APS, Pisa, Italy; 3 Health Psychology, ISPA, Lisboa, Portugal

**Keywords:** social isolation, Health Survey, COVID-19, Life satisfaction

## Abstract

**Introduction:**

Although some philosophers recognize in the loneliness an evolutionary existential process, a 2019 declaration of World Health Organization underlines the major health problem in the worldwide is the perception of state of loneliness. The feeling of loneliness linked to the social isolation (SI) or a lack of social opportunity activate a stressful condition associated to an increase of social dependence. This ‘learned social helplessness’ can be dangerous so that it is associated with an increased prevalence of suicides (Cacioppo and Cacioppo, 2018; Bzdok and Dunbar, 2020). Considering the impact of loneliness on the mental health we can assume that the COVID-19 forced SI affects the state of health and psychosocial well-being.

**Objectives:**

To evaluate the psychosocial impact of the SI in Italy.

**Methods:**

An ad hoc survey have been sent from May to June 2020.

**Results:**

These results refer to the Italian survey of a multicenter investigation with partnership of Spain and Portugal universities. The investigation is in progress being a longitudinal study. Of the total 292 subjects investigated (age xM: 34; sD14.13), 118 (40,41%) had been in SI. Subjects forced into SI report more interference in the life satisfaction (p=0.003) though no more anxiety, depression and hostility we found in the SI group.

**Conclusions:**

During the phase 2 of Italian COVID-19 diffusion, we found an impact on the life satisfaction more than psychopathology. We can assume that the impact of mental health it may occur as the reduction in life satisfaction associated with forced SI continues.

